# Clinical Outcome in Elderly Head and Neck Cancer Patients Treated with Concomitant Cisplatin and Radiotherapy

**DOI:** 10.3390/cancers17183007

**Published:** 2025-09-15

**Authors:** Chiara Lucrezia Deantoni, Andrea Galli, Davide Valsecchi, Luca Porcu, Lucrezia Tranò, Laura Giannini, Italo Dell’Oca, Anna Chiara, Vittorio Gioffrè, Moreno Tresoldi, Nadia Gisella Di Muzio, Leone Giordano, Aurora Mirabile

**Affiliations:** 1 Department of Radiation Oncology, IRCCS San Raffaele Scientific Institute, 20132 Milan, Italy; deantoni.chiaralucrezia@hsr.it (C.L.D.); giannini.laura@hsr.it (L.G.); delloca.italo@hsr.it (I.D.); chiara.anna@hsr.it (A.C.); dimuzio.nadia@hsr.it (N.G.D.M.); 2 Department of Otorhinolaryngology - Head and Neck Surgery, IRCCS San Raffaele Scientific Institute, via Olgettina 60, 20132 Milan, Italy; trano.lucrezia1@hsr.it (L.T.); gioffre.vittorio@hsr.it (V.G.); giordano.leone@hsr.it (L.G.); mirabile.aurora@hsr.it (A.M.); 3 Faculty of Medicine and Surgery, Vita-Salute San Raffaele University, via Olgettina 58, 20132 Milan, Italy; 4 Emergency Department, IRCCS San Raffaele Scientific Hospital, 20132 Milan, Italy; valsecchi.davide@hsr.it; 5 Cancer Research UK Cambridge Institute, University of Cambridge, Cambridge CB2 1TN, UK; luca.porcu@cruk.cam.ac.uk; 6 General Medicine and Advanced Care Unit, IRCCS San Raffaele Scientific Institute, 20132 Milan, Italy; tresoldi.moreno@hsr.it

**Keywords:** head and neck, radiotherapy, chemotherapy, elderly, geriatric assessment

## Abstract

Background: Cisplatin (CDDP) concomitant to radiotherapy (RT) is one of the main treatments for locally advanced head and neck squamocellular carcinoma (LA HNSCC); nevertheless, elderly patients are underrepresented in trials and frequently receive less intensive and suboptimal treatments, which often are unimodal, even if it is well demonstrated how chemoradiotherapy (CRT) with CDDP is superior to RT alone. We aim to analyze clinical feasibility in terms of acute toxicity and treatment adherence in patients receiving concurrent high-dose CDDP and RT with curative or adjuvant intent, comparing elderly (≥65 years) with young (<65 years) patients. Methods: This is a monocentric, observational, prospective study of consecutive LA HNSCC patients treated with high dose CDDP concomitant to RT, from January 2017 to June 2024. The primary aim is to compare the performance of elderly patients with young patients in terms treatment adherence and toxicity, while the secondary aim is to assess treatment efficacy in terms of OS and PFS endpoints. The LA HNSCC patients were selected only by treatment, so all patients who did not receive chemoradiation were excluded. No difference in terms of compliance and toxicity events >G1 in the elderly versus young population is the null hypothesis. Data were analyzed using MedCal statistical software 14.12. A *p* < 0.05 was considered statistically significant. Results: A total of 170 patients were included in the study, according to the selection criteria stated. Only 7 elderly (12.3%) patients received a dose < 200 mg/m^2^, whereas 163 patients (87.7%) received ≥ 200 mg/m^2^ and all elderly patients completed RT, indicating a high level of adherence and effective management of treatment protocols, in front of a comparable incidence of acute toxicity to young patients (*p*-value: 0.84). OS and PFS were not statistically different between elderly and young patients (*p* = 0.20 and *p* = 0.72, respectively). Conclusions: Our findings suggest the feasibility of curative oncological treatment for elderly individuals with LA HNSCC, if they are well selected and adequately supported. Future studies should focus on validating and expanding these results to improve patient care and outcomes.

## Simple Summary

In elderly patients with locally advanced head and neck tumors, chemotherapy and radiotherapy are often de-escalated due to concerns about side effects, potentially compromising treatment efficacy. This study demonstrates that, with careful patient selection and appropriate supportive care during treatment, the toxicity profile of chemoradiotherapy in elderly patients is comparable to that observed in younger individuals.

## 1. Introduction

Head and neck squamous cell carcinomas (HNSCC) are the sixth most prevalent malignancy globally, accounting for approximately 6% of all newly diagnosed cancer cases. Although there is a rising incidence of human papillomavirus (HPV)-associated cancers in younger individuals, around 25–40% of patients are aged over 70 years [1].

Elderly individuals have often been excluded or underrepresented in clinical trials assessing the effectiveness of radiotherapy or chemotherapy for HNSCC, making it difficult to generalize the results to this population [2,3,4,5].

According to the consensus definition of the United States National Institute of Aging, elderly patients could be categorized into “young elderly” (65–74 years), “older old” (75–84 years), and “oldest old” (>84 years) [6]. However, the correlation between age and treatment outcomes suggests that chronological age is frequently less significant than biological age. Still, multiple demographic studies indicate that while nearly 90% of patients under 60 years receive standard-of-care treatment, only about 60% of those over 70 years are managed according to clinical guidelines [7], even though quality of life and survival rates in older patients undergoing curative therapies are comparable to those of younger patients, based on retrospective analyses [8].

Frequently, older subjects receive less intensive and suboptimal treatments, which often are non-surgical and unimodal [1], even if it is well demonstrated how chemoradiotherapy with cisplatin or bio-radiotherapy with cetuximab are superior to RT alone [5,9].

In fact, the use of concomitant chemoradiotherapy (CRT) showed an improvement in overall survival of 6.5% at 5 years compared to radiotherapy alone, but only in patients under 70 years old [10,11]. A complete response was identified in 27.4% in RT alone, and 40.2% in CT/RT; the 3-year projected disease-specific survival was 33% in RT alone vs. 51% for CT/RT [2].

However, the role of CRT in older people remains unclear because they could be more prone to develop treatment-related toxicities, jeopardizing their prognosis and quality of life. Indeed, comorbidities are significantly more present in elderly HNSCC patients, thus limiting surgical approaches and CRT. In an outpatient oncology clinic, the following conditions and their prevalence were observed in older cancer patients [12]: comorbidities in over 90% of patients (30–40% of severe degree), dependence on others due to functional status measured by Instrumental Activities of Daily Living (IADL) in 50–60%, nutritional impairment in 30–50%, depression in 20–40%, and cognitive impairment in 25–35%. That considered, approximately half of the patients aged over 70 years could be treated with the oncological standard of care. Clinical features and treatment-related toxicities were largely comparable between older and younger patients, with the exception that older individuals experienced greater weight loss, had a higher need for feeding tube assistance, and were more prone to developing grade 3 or higher hematologic toxicities and sepsis during the course of chemoradiotherapy. Aside from overall survival, which was influenced by the age disparity between the two groups, the disease-specific outcomes, including disease-free survival and cancer-specific survival, were remarkably similar across both age cohorts [13].

When considering older patients specifically, the balance between the likelihood of controlling the tumor (the tumor control probability) and the expected toxicities from the treatment (the therapeutic ratio) is much more delicate. In other words, although the treatment may be effective in eradicating the tumor, in older patients, the risk and severity of side effects may offset the benefits [14].

The aim of this study is to investigate whether older adults are more prone to experiencing higher levels of toxicity and reduced compliance, conditions that may render them ineligible for chemoradiotherapy.

## 2. Patients and Methods

### 2.1. Study Design

The study is a monocentric, observational, and prospective investigation conducted in an a tertiary academic referral center. Data were collected from a specific group of patients (aged ≥ 18 years), Eastern Cooperative Oncology Group (ECOG) performance status 0–1, suffering from locally advanced squamous cell carcinoma of the head and neck, including tumors arising in the salivary glands and thyroid gland. Patients included in this study underwent either surgery followed by adjuvant CRT or radical, upfront CRT with curative intent. Subjects with general contraindications to chemotherapy (GFR < 60 mL/min, cardiac ejection fraction < 60 mL/min, neuropathy, hearing loss, Child B-C liver failure), or submitted to treatment with non-curative intent were excluded.

The study time frame was from January 2017 to June 2024.

Assuming at least 30% of elderly patients, 170 patients are necessary to statistically detect the following scenarios, with a type I error of 5% and a two-tailed test:

1. An increase of 30% of non-compliant elderly versus young patients with a statistical power of 87%. A percentage of non-compliant young patients equal to 10% was assumed.
2. An increase of 25% mucositis grades > 2 in elderly versus young patients with a statistical power of 86%. A percentage of young patients with toxicity grade > 2 equal to 30% was assumed.
3. An increase of 25% dysphagia grades > 2 in elderly versus young patients with statistical power of 85%. A percentage of young patients with toxicity grade > 2 equal to 35% was assumed.
4. An increase of 25% dermatitis grades > 2 in elderly versus young patients with a statistical power of 87%. A percentage of young patients with toxicity grade > 2 equal to 25% was assumed.

Sample size calculation was performed using pwrss R package Version 4.5.0.

The primary aim was to assess the feasibility of CRT in terms of side effects associated with these curative strategies and treatment adherence, comparing elderly to young patients. No difference between the two groups is the null hypothesis.

OS and PFS endpoints, defined, respectively, as the duration of time from cancer diagnosis to the time of death from any cause and the length of time from treatment initiation (surgery or chemoradiation) to the first occurrence of disease progression or death, were used as secondary endpoints to assess treatment efficacy. Disease progression was defined by follow-up imaging (MRI, CT, PET-CT) or, in selected cases, by biopsy.

### 2.2. Patients’ Characteristics and Data Collection

Patients’ general characteristics were collected, including age, gender, stage (according to AJCC 8th edition [15]) and anatomical site of the primary tumor, lifestyle habits, and performance status according to ECOG scale. Further, Adult Comorbidity Evaluation 27 (ACE-27) and Geriatric 8 (G8) scores were recorded.

Surgery details and techniques were also registered, as well as the main parameters of the intensity modulated radiotherapy, such as the date of first and last fraction, total dose, dose per fraction, clinical target volumes, and interruptions longer than three days. All patients were treated with helical TomoTherapy^®^ (HT-Accuray, Maddison, WI, USA). According to clinical guidelines, the chemotherapy regimen consisted of cisplatin (CDDP) administered at a dosage of 100 mg/m^2^ every three weeks for a total of a minimum of 2 and maximum of 3 cycles; this was combined with the radiotherapy protocol lasting 6–7 weeks. All patients were regularly evaluated during their follow-up period with clinical and instrumental examinations. Eventual tumor recurrence was classified as local, regional, or distant according to the site of presentation. Local relapses were also classified as in-field (if recurrence occurred within the previous radiotherapy volume) or out-field.

Acute and late events associated with the curative treatment course were evaluated using the Standard Common Criteria for Adverse Events (CTCAE) v4.1 [16].

### 2.3. Statistical Analysis

Nonparametric statistics were employed to outline the key characteristics of the study population. This involved calculating medians and interquartile ranges (IQR) for continuous variables, as well as frequencies and proportions for categorical variables.

The Kaplan–Meier approach was utilized to assess overall survival (OS) and progression-free survival (PFS). Comparisons between survival curves were made using the log-rank test.

Analysis was performed considering all consecutive patients treated with chemoradiation and comparing elderly (or ≥ 65 years) to young patients (<65 years). Toxicities of the two groups (i.e., elderly vs. young population) were compared using the logistic regression model. Odds ratios were estimated, and Wald tests were performed to detect statistical association,

A subgroup analysis was performed in elderly individuals comparing patients treated with surgery followed by adjuvant chemoradiotherapy (surgery + CRT) to those treated with radical chemoradiotherapy (CRT), using the log-rank test.

Data were analyzed using MedCal statistical software version 14.12.

A *p* < 0.05 was considered statistically significant.

### 2.4. Ethical Statement

This prospective observational study was designed in accordance with the principles established by the Eighteenth World Medical Assembly (Helsinki, 1964) and all pertinent amendments from the World Medical Assemblies and the ICH Guidelines for GMP. The study received institutional Ethical Committee approval.

## 3. Results

### 3.1. Patients’ Characteristics

A total of 170 consecutive patients were included in the study, according to the stated selection criteria. The main characteristics of the sample are listed in Table 1.

The population was divided into Elderly (>65 years old) and Young (<65 years old).

The median age of elderly patients was 72 years (IQR 70–75). The age distribution shows that 21% of the patients were between 65 and 69 years old, while the majority (78.9%) were aged 70 years or older.

For the young patients, the median age was 57 years (27–65).

Performance status, as measured by the PS-ECOG scale, shows that 59.6% of the patients had a score of 0 (versus 73.5% in the control arm, *p*-value = 0.23), while 40.4% had a score of 1 (versus 26.5% in the control arm, *p*-value = 0.44).

Most of the elderly patients (73.7%, 42 patients) were treated with upfront CRT, whereas 26.3% (15 patients) were treated with surgery followed by adjuvant CRT. Considering the two mentioned subgroups (surgery + CRT versus radical CRT), no significant differences were found in tumor stage, ECOG PS, or G8 score. In the control arm, 80 patients (70.8%) underwent radical CRT, and 33 patients (29.2%) were treated with adjuvant CRT.

In the radical chemoradiotherapy (CRT) setting, 18-Fluorodeoxyglucose positron emission tomography (PET) combined with computed tomography (CT) was utilized to delineate the biological target volume (BTV). These patients received a hypofractionated regimen with a simultaneous integrated boost—54 Gy in 30 fractions to bilateral neck lymph nodes and 66 Gy to the primary tumor and high-risk/PET-positive nodes [2,17,18].

In the adjuvant CRT setting, radiotherapy dosing was guided by histopathological findings: 54 Gy in 30 fractions was delivered to low-risk regions, while 61.5 to 64 Gy in 30 fractions was administered to high-risk areas [3,4,19,20].

The analysis of the total CDDP dose administered to the elderly revealed that only seven patients (12.3%) received a dose lower than 200 mg/m^2^, whereas most of them (87.7%) received a dose equal to or greater than 200 mg/m^2^. All patients in the “young” group received a dose equal to or greater than 200 mg/m^2^.

Impressively, all elderly patients completed their radiotherapy (RT) treatment, indicating a high level of adherence and effective management of treatment protocols. However, a high incidence of acute toxicity was found to be grade ≥ 2 toxicity, which included dermatitis in 34 patients (59.6%), mucositis in 24 patients (42.1%), and dysphagia in 21 patients (36.8%). In the control arm, the incidence of acute toxicity was comparable (*p*-value: 0.82; Table 2). Delayed toxicity was less frequently reported, with only two cases of G2 xerostomia and one patient with G2 fibrosis.

### 3.2. Survival Analysis

**Figure 1:**
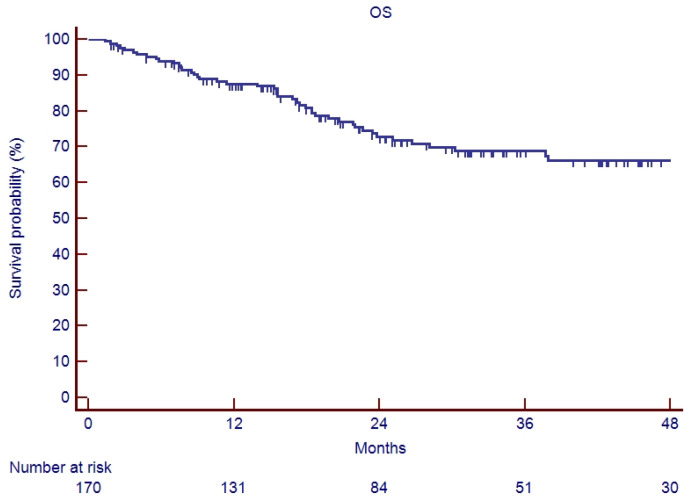
Overall survival of study sample.

**Figure 2:**
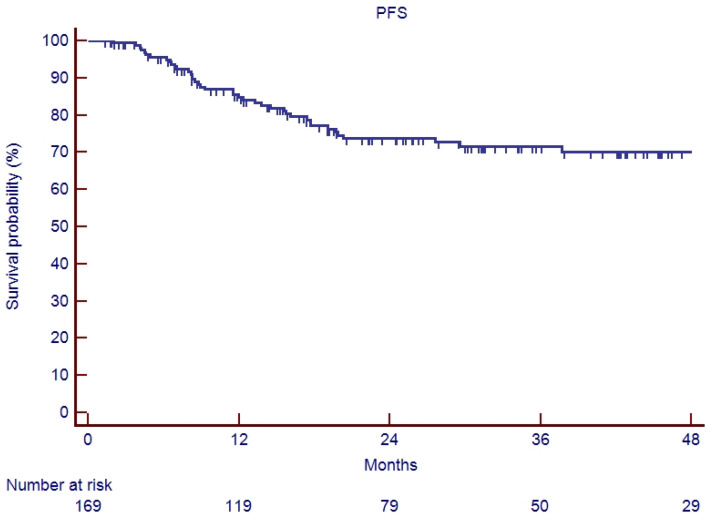
Progression-free survival of study sample.

**Figure 3:**
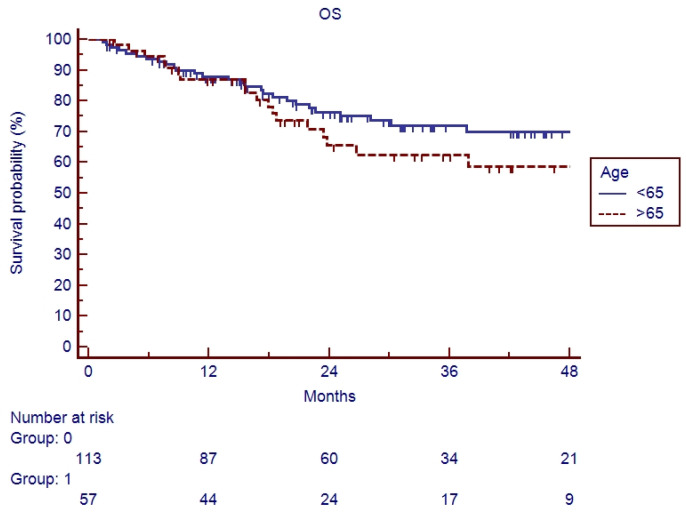
Overall survival of elderly vs. young patients.

**Figure 4:**
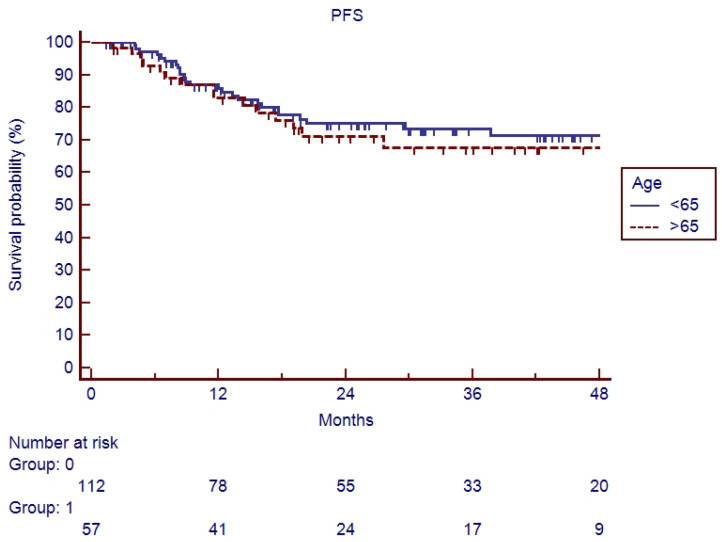
Progression-free survival of elderly vs. young patients.

**Figure 5:**
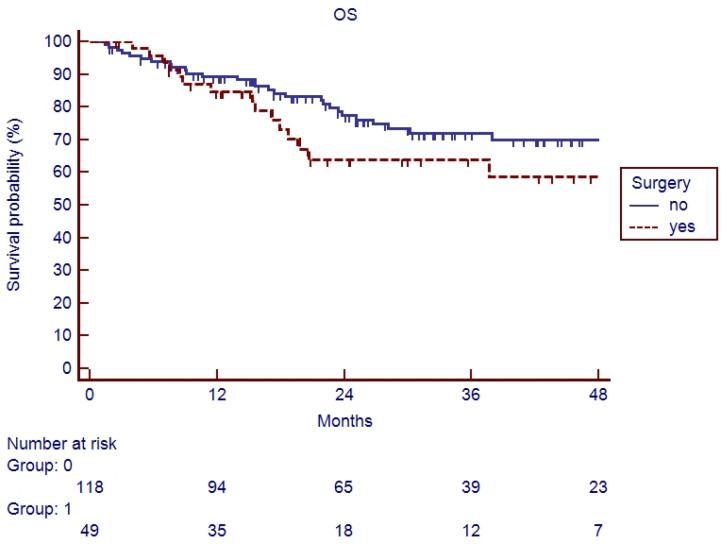
Overall survival of patients undergoing surgical treatment vs. CTRT.

**Figure 6:**
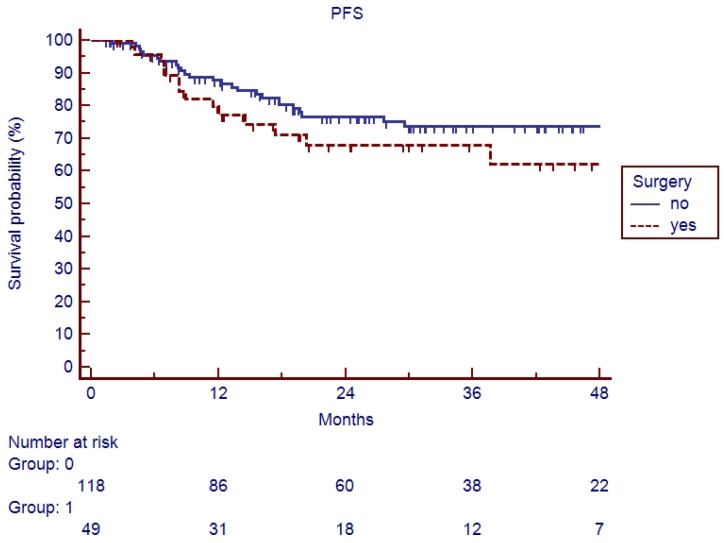
Progression-free survival of patients undergoing surgical treatment vs. CTRT.

In the elderly patients, the median follow-up was 20.6 months (range 2–90). Fifteen patients (26.3%) experienced a relapse during their follow-up: eleven recurrences were judged as out-field with four in-RT-field. OS and PFS of the whole cohort of patients are represented in Figure 1 and Figure 2. Indeed, 1-year, 2-year, and 3-year OS were 86.9%, 65.5%, and 62.5%, respectively (Figure 1), and 1-year, 2-year, and 3-year PFS were 82.9%, 71.1%, and 67.7%, respectively (Figure 2). Overall survival and PFS were not statistically different between elderly and young patients (Figure 3 and Figure 4; *p* = 0.20 and *p* = 0.72, respectively) or between surgery + CRT and CRT (Figure 5 and Figure 6; *p* = 0.47 and *p* = 0.26, respectively).

## 4. Discussion

The available literature about elderly patients affected by head and neck cancer highlights the complexities and challenges associated with curative treatment in this age group. In fact, despite the increasing involvement of older patients in cancer care, this demographic is still significantly underrepresented in clinical trials, primarily due to pre-existing medical conditions that make them ineligible regardless of their diagnosis. This lack of representation persists as an ongoing issue, even though these patients often express a willingness to participate [21].

Currently, only about 3.4% of studies are focused on patients over 65 years of age. This situation represents a significant gap in our understanding of their specific healthcare needs, and more importantly, in the development of effective treatments for this age group [22].

As stated, elderly patients often have multiple comorbidities, which greatly affect their possibilities to undergo comprehensive treatment strategies. Research shows that surgery may carry a higher risk of morbidity and mortality in this age group, especially those with multiple disorders in their medical history [23]. Also radical CRT may be difficult for elderly individuals, potentially leading to treatment interruptions, reduced tolerance to standard regimens [1], compromised outcomes, and diminished quality of life. Therefore, treatment modifications, such as altered fractionation schedules in radiation, lower chemotherapy doses, and improved supportive care, are often necessary. In particular, providing supportive care is crucial for these fragile patients to complete their designated treatment plan without interruptions and to safely recover from harmful effects. Specifically, it has been observed that keeping a good nutritional status improves the patient’s ability to tolerate the treatment [24] and results in comparable disease-related survival rates when compared to younger patients [13].

In our series, all patients included had a PS- ECOG score of either 0 or 1, indicating good to excellent performance status. This observation underscores a fundamental principle in the management of elderly patients with HNSCC: patients who are candidates for concomitant CRT are necessarily those with the best performance status. This selection bias is not just incidental but is in fact crucial for several reasons.

Concomitant CRT is an intensive treatment regimen that places significant physiological stress on patients. Those with poorer performance status may not be able to tolerate the combined acute and delayed toxicities of chemotherapy and radiation.

It is evident that every intervention (surgery, radiotherapy, chemotherapy) may adversely affect quality of life (QoL) [25,26]. Nowadays QoL assessment has become more preeminent, emphasizing the association between patients’ perception and therapeutic outcomes [27].

By selecting patients with good performance status, the likelihood of completing the planned treatment course is increased, which is crucial for achieving optimal oncological outcomes.

In elderly patients, the potential benefits of an aggressive treatment such as CRT must be carefully weighed against the risks of treatment-related morbidity and mortality. Good performance status helps tip this balance in favor of more intensive treatment.

Patients with better baseline performance are more likely to maintain a reasonable quality of life during and after treatment, which is particularly important in the elderly population.

The question of whether age should be a determining factor for irradiation was brought to the forefront of research over two decades ago. A notable study led by Pignon and his team, titled “No age limit for radical radiotherapy in head and neck tumors”, initiated this discussion. This significant meta-analysis pooled data from 1589 patients, of which 26% were over 65 years old, from five EORTC trials [18,19,28].

To analyze the data, the research team divided the patient database into seven age categories, three of which were classified as part of the elderly population (65–69, 70–75, and over 75). Their findings were quite revealing as there were no notable differences in overall survival, locoregional control, acute objective mucosal reactions, weight loss, or late effects across the different age groups. However, when considering functional mucosal reactions, older adults appeared to experience these effects more frequently. This age dependency disappeared once adjustments were made for performance status, suggesting that it was not age per se, but rather the physiological condition of the individual, that influenced the incidence of these reactions.

This led the researchers to hypothesize that elderly people might experience more difficulty in tolerating acute toxicity than their younger counterparts, potentially due to underlying health conditions or decreased physiological resilience.

In conclusion, the authors of the study argued that chronological age should not be the primary determinant in therapeutic decisions. Instead, patients’ individual health status and ability to tolerate treatment should be considered. However, it should be noted that subjective acute toxicity and lower tolerance to treatment do appear to be more common in elderly patients, indicating that this demographic may require more careful management and monitoring during treatment [28].

These findings align with our observations, and notably, the rates of objectively assessed acute mucositis showed no significant differences in terms of grade or duration.

A more recent retrospective analysis [29] indicated that both radiotherapy and chemoradiotherapy (CRT) are viable treatment options for older patients with good performance status, demonstrating high locoregional control rates. However, the median overall survival (OS) of 34 months in this elderly cohort was inferior to outcomes observed in younger, more selectively chosen populations in large randomized controlled trials, such as RTOG 9501 (with a median OS 44.9 months) [3] and EORTC 22931 (median OS 72 months) [4]. The toxicity profile was unfavorable, with 56.1% of patients experiencing grade 3–4 acute toxicities according to CTCAE criteria.

In the review by Chang [13], treatment-related toxicities appeared comparable between older and younger patients. However, the elderly population was more susceptible to weight loss, need for feeding tube support, and grade ≥3 hematologic toxicity and sepsis during CRT. Despite these challenges, disease-related outcomes—including disease-free and disease-specific survival—were largely similar between age groups.

Another retrospective, single-center analysis [30,31] did not report significant differences in treatment interruption rates, completion rates, or treatment-related mortality between younger and older patients undergoing non-surgical modalities such as CRT. These observations support our findings and reinforce the practicality of such treatment strategies in the elderly.

Age and performance status remain key prognostic indicators. However, due to the subjective nature of performance status assessments, the implementation of objective geriatric evaluations has been recommended to enhance patient selection.

The literature suggests the use of comprehensive geriatric assessments (CGAs) to better personalize treatments for each patient and improve outcomes [11]. Still, evidence regarding their impact specifically in the head and neck squamous cell carcinoma (HNSCC) setting remains scarce. Preliminary results show that CGA influenced treatment decisions in approximately 10% of cases (8% in ELAN ONCOVAL study [32] and 11.8% in ELDERLY study [33]). While no definitive data confirm that CGA improves outcomes in HNSCC, many experts advocate for its routine implementation prior to treatment. Nevertheless, the widespread use of CGA is often hindered by its complexity and time requirements. To address this, shorter screening tools have been introduced to identify high-risk patients who might benefit from full geriatric evaluation. Among them, the G8 screening tool is the most commonly used and has shown good concordance with CGA results [34]. In this study, we incorporated the G8 score into our patient classification system to better assess the influence of age-related vulnerability on treatment outcomes and to personalize therapeutic strategies accordingly.

Neve et al. found that an impaired G8 score correlated with poorer postoperative outcomes and lower treatment completion rates in the CRT group [35]. An Italian AIRO survey investigating care for elderly HNSCC patients revealed that, although multidisciplinary team discussions are standard practice, a geriatrician is seldom involved, and CGA is performed in only 10% of radiotherapy and oncology centers. This shortfall increases the risk of inappropriate treatment decisions due to inaccurate frailty assessments [36]. In our department, it is customary to send patients older than 65 years to a geriatrician for a complete evaluation before treatment decision. In this series, 74.5% of patients achieve a G8 score ≥15, indicating our attempt to select patients with multimodal evaluation.

Looking at the data, we found that only a small group of patients, specifically 7 (roughly 12.3% of the total) out of 57 patients enrolled in the study, received a total CDDP dose lower than the 200 mg/m^2^ mark. Al-Mamgani et al. demonstrated that a cumulative CDDP dose below this threshold may negatively impact outcomes and should be avoided in clinical practice. Their study reported significantly worse OS, DFS, locoregional control (LRC), cancer-specific survival, and distant metastasis-free survival (DMFS) among patients who either received radiotherapy alone or a cumulative CDDP dose under 200 mg/m^2^, regardless of the specific dosing regimen (e.g., daily low-dose or single-cycle administration), compared to those who received ≥ 200 mg/m^2^ (via two or three treatment cycles) [37]. This deviation from the standard dosage in 7/57 patients of our series is likely related to patients’ specific comorbidities, a correlation supported by the ACE-27 score findings. We observed that five out of seven of these patients were classified as grade 3 for comorbidities, indicating severe or multiple health problems. Additionally, two out of seven of these patients had a G8 score < 15.

In our study, only 15 patients underwent surgical treatment followed by CRT, representing a small subset of the overall patient population (57 patients), which certainly does not allow for any treatment comparison. This deserves careful consideration. The decision to analyze operated and non-operated patients in the same series stems from the assumption that, in any setting when deciding on the best treatment, patient selection is crucial. Patients who did not demonstrate a sufficient PS after surgery were excluded from the analysis. On the other hand the characteristics of the surgical group reveal a highly selected patient population. The majority (73.3%) are over 70 years old, yet they were deemed fit for surgery. The PS-ECOG scores (46.7% at 0 and 53.3% at 1) suggest that only patients with good functional status were considered for surgery. Despite the advanced age and comorbidities, 93.3% of surgical patients received ≥ 200 mg/m^2^ total cisplatin dose in their adjuvant CRT. This high rate of treatment completion suggests careful patient selection in the surgical cohort, adequate supportive care, and good tolerance of the multimodality approach.

For the evaluation of survival endpoints, we considered all patients, with the awareness however that a small percentage have histologies with a different prognosis compared to squamous cell carcinomas (for example, thyroid, paranasal sinuses). Moreover, our OS results are comparable to the ones obtained by Cooper et al. [4] in postoperative concurrent CRT for high-risk squamocellular carcinoma of head and neck, in which 1-year, 3-year, and 5-year OS were about 80%, 60%, and 45%, respectively, regardless of age. In contrast, regarding DFS, the findings of Cooper et al. were worse than ours, reporting 1-year, 3-year, and 5-year DFS of about 65%, 40%, and 30%, respectively, regardless of age.

Also in a definitive not-surgical setting, our findings proved better than the literature, where, for example, Haehl et al. [29], in 2020, evaluated the outcome in geriatric head and neck cancer patients undergoing chemoradiation or radiotherapy alone reported 2-year rates for OS and PFS of 56.9 and 44.9%, respectively. Interestingly, low performance status (HR = 2.584, 95% CI 1.561–4.274; *p* < 0.001) and smoking (HR = 1.960, 95% CI 1.109–3.464, *p* < 0.05) were the strongest independent prognostic factors affecting OS in their multivariate analysis.

In conclusion, caring for patients requires nowadays a multidimensional strategy with a comprehensive assessment taking into account the age of the patients and their pre-existing medical conditions. This underscores the need for a multidisciplinary approach involving otolaryngologists, oncologists, radiation oncologists, geriatricians, nutritionists, and other specialists to address the heterogeneous health issues that usually affect elderly cancer patients. In any case, CRT for older head and neck cancer patients should be performed with maximum supportive care [38] and preferably in high-volume centers [39]. Managing older patients during chemoradiotherapy requires careful assessment and close monitoring, trying to prevent and recognize side effects as soon as possible. A twice-a-week clinical examination is often performed by expert-in-field radiation oncologist or medical oncologist, with a close collaboration with the dedicated nutritionist.

Our findings add to the growing evidence that elderly patients are capable of tolerating intensive, curative treatments without experiencing a significantly greater decline in quality of life compared to younger individuals [40,41]. The greatest risk, in fact, lies in the potential for undertreatment of this population [42].

## 5. Conclusions

Older adults with HNSCC still remain a relevant clinical issue. Comorbidities significantly impact treatment decisions, especially when dealing with surgical refinements, altered radiotherapy schedules, and adjusting chemotherapy doses. In these settings, supportive care measures, including nutrition and hydration, and multidimensional assessment become critical. Indeed, the decision on whether to treat a patient cannot be based solely on age, because this could exclude patients from treatment that they are able to tolerate and benefit from. It is essential to adopt a more holistic approach in treatment decisions to ensure all patients, regardless of age, are given every opportunity to benefit from potential treatment responses. Our findings underline the feasibility of curative oncological treatment also for elderly patients with HNSCC, if they are well selected and adequately supported. Future studies should focus on validating and expanding these results to improve patient care and outcomes.

## Figures and Tables

**Table 1:** Characteristics of elderly and young patients.

Characteristics of Included Patients	Elderly (57 Patients)	Young (113 Patients)
Age, years, median [IQR]	72 [70–75]	57 [27–65]
Age class		
>65	12 (21.1%)	NA
>70	45 (78.9%)	NA
Gender		
Male	42 (73.7%)	86 (24.0%)
Female	15 (26.3%)	27 (76.0%)
Adult Comorbidity Evaluation (ACE)-27 levels		
Grade 1	6 (10.5%)	NA
Grade 2	36 (63.2%)	NA
Grade 3	15 (26.3%)	NA
Smoking		
>10 packs/year	38 (66.7%)	55 (48.7%)
<10 packs/year	6 (10.5%)	20 (17.7%)
No	13 (22.8%)	38 (33.6%)
Alcohol		
≥500 mL	5 (8.8%)	12 (11.0%)
No or <500 mL	52 (91.2%)	101 (89.0%)
Drug abuse		
Yes	0	0
No	57 (100.0%)	113 (100.0%)
ECOG Performance Status Scale		
0	34 (59.6%)	83 (73.5%)
1	23 (40.4%)	30 (26.5%)
Tumor staging		
I	0	4 (3.5%)
II	2 (3.6%)	10 (8.8%)
III	14 (24.6%)	41 (36.3%)
IV	41 (71.9%)	58 (51.4%)
Tumor location		
Oral cavity	9 (15.8%)	16 (14.2%)
Oropharynx	23 (40.3%)	52 (46.0%)
Hypopharynx	5 (8.8%)	8 (7.1%)
Larynx	9 (15.8%)	8 (7.1%)
Nasopharynx	2 (3.5%)	17 (15%)
Nasal cavity	4 (7.02%)	4 (3.5%)
Paranasal sinuses	1 (1.7%)	0
Salivary glands	3 (5.3%)	7 (6.2%)
Thyroid	1 (1.7%)	0
External auditory canal	0	1 (0.1%)
G8 score, *n* (%)		
≥15	43 (75.4%)	NA
<15	14 (24.6%)	NA

**Table 2:** Toxicity.

	Elderly	Young	Odds Ratio	*p*-Value
Dermatitis				
G0–1	23 (40.4%)	52 (44.4%)	1	0.609
G ≥ 2	34 (59.6%)	65 (55.6%)	1.18	
Mucositis				
G0–1	33 (57.9%)	55 (47.1%)	1	0.179
G ≥ 2	24 (42.1%)	62 (52.9 %)	0.65	
Dysphagia				
G0–1	36 (63.2%)	72 (61.5%)	1	0.836
G ≥ 2	21 (36.8%)	45 (38.5%)	0.93	

Note: Logistic regression model was used to estimate odds ratios and detect statistical association. Wald tests were used to detect statistical association.
